# Connecting Caries to Cause: Unraveling the Impact of Socioeconomic and Demographic Factors on Early Childhood Caries

**DOI:** 10.7759/cureus.90490

**Published:** 2025-08-19

**Authors:** Surbhi Sharma, Meenakshi Sharma, Shriya Gupta, Hage Monju, Nanmaran P N, Neel Chaudhary

**Affiliations:** 1 Department of Pedodontics and Preventive Dentistry, Pacific Dental College and Hospital, Debari, Udaipur, IND; 2 Department of Pedodontics and Preventive Dentistry, Rajasthan University of Health Sciences College of Dental Sciences, Jaipur, IND; 3 Department of Pedodontics and Preventive Dentistry, Government College of Dentistry, Indore, IND; 4 Department of Pedodontics and Preventive Dentistry, Ragas Dental College and Hospital, Uthandi, Chennai, IND

**Keywords:** demographic factors, early childhood caries (ecc), oral hygiene status, risk factors, socio-economic factors

## Abstract

Introduction

Early childhood dental health plays a crucial role in a child's overall well-being and development. Maintaining healthy teeth is essential for optimal eating and speaking functions, while also contributing to a positive appearance that encourages social interactions. In recent years, significant attention has been given to examining the connection between childhood tooth decay, socioeconomic status, and various other influencing factors.

Aim

The study aimed to find out the association between early childhood caries and the socioeconomic status of the children.

Methodology

This study examined 260 children aged 3 to 6 years. After being diagnosed with early childhood caries, the children's parents completed a questionnaire. Demographic data were recorded, and dental assessments followed WHO guidelines. Socioeconomic status was evaluated using the Kuppuswamy and Udai Pareekh scales.

Results

The prevalence of early childhood caries (ECC) in the study population was 34.6% (n = 90), while 65.3% (n = 170) remained caries-free. ECC was more common among children from lower socioeconomic backgrounds (66.7%). Risk factors included on-demand breastfeeding, nighttime bottle feeding, and frequent snacking between meals. Conversely, early initiation of toothbrushing, higher brushing frequency, use of toothbrushes, and fluoridated toothpaste were identified as protective factors against ECC.

Conclusion

The study revealed a significant association between the identified risk factors and early childhood caries (ECC). These results emphasize the need to address socio-demographic and lifestyle influences as part of effective strategies for preventing and managing dental caries in children.

## Introduction

For a child's general health and well-being, early childhood dental health is crucial [1]. Early dental hygiene and health practices promote good oral health throughout infancy, adolescence, and maturity [2]. According to epidemiological research, dental caries is the most common chronic illness affecting children worldwide and costs healthcare systems an enormous amount of money. Early childhood caries risk factors must be identified because research indicates a high correlation between the overall condition of primary teeth and the health of young permanent teeth [3].

The status of primary teeth has a strong correlation with the health of permanent dentition, underscoring the importance of identifying and addressing risk factors for caries in early childhood [3]. The American Academy of Pediatric Dentistry (AAPD) defines early childhood caries (ECC) as the presence of one or more decayed (non-cavitated or cavitated), missing (due to caries), or filled surfaces in any primary tooth in a child under 71 months of age. Severe ECC is characterized by more extensive involvement, such as smooth surface lesions or decay in anterior teeth, depending on the child’s age [4].

ECC impacts young children globally [5]. Several studies conducted in southern India have reported varying caries prevalence among preschool children, with rates ranging from 25% to 70% [6]. It is regarded as a significant oral health concern, particularly in disadvantaged communities of both developing and industrialized nations, where undernutrition is prevalent [7].

The development of ECC is believed to follow a multifactorial disease model, with psychosocial risk factors like maternal mental health issues, lack of education, and poverty playing an increasingly important role [8]. Risk factors for dental caries in preschoolers can be classified into biological, environmental, and socio-behavioral categories. High consumption of sucrose, sugary beverages, frequent intake of sugar-rich foods between meals, and regular snacking have all been associated with an increased risk of dental caries.

India is a vast country having different cultures and socioeconomic strata with diverse behavioral traditions. All these factors have a direct bearing on oral health [9]. The socioeconomic status (SES) is one of the important factors affecting the health condition of an individual or a family [10]. It has been shown that a child’s oral hygiene patterns are influenced by socioeconomic conditions and parents' oral health behaviors [11-14].

Currently, there is no data available regarding the prevalence of caries and its association with other factors among preschool children in Jaipur district, Rajasthan. Therefore, this study aimed to evaluate via a questionnaire the association of early childhood caries with BMI and the socio-economic status of the family among 3- to 6-year-old children.

## Materials and methods

Study design and population

A cross-sectional survey was conducted for one year from 1.9.2021 to 31.8.2022 and included a total of 260 children aged 3 to 6 years. Participants were selected using convenience sampling from among children visiting the Outpatient Department of Pedodontics and Preventive Dentistry at RUHS College of Dental Sciences, Jaipur, Rajasthan.

The sample size was calculated based on the following equation:

Sample size = $\frac{Z^2 \times p \times (1-p)}{e^2}$ where $Z$ = critical value of the normal distribution at the required confidence level, $p$ = sample proportion, and $e$ = margin of error (5%); therefore, $n = \frac{(1.96)^2 \times 0.20 \times 0.80}{(0.05)^2} = 246$.

Thus, the minimum sample size required was calculated to be approximately 246.

To account for a possible 10% non-response or attrition rate, the sample size was inflated:

Adjusted sample size = $\frac{246}{0.90} \approx 273$.

However, due to logistical and operational feasibility, the final sample size was set at 260 children aged 3-6 years, which still meets the power requirements for the study.

The assumed prevalence of 20% was based on previous studies assessing ECC prevalence in similar populations within India [5].

Inclusion and exclusion criteria

Children within the age range of 3 to 6 years who attended the department during the study period were included. Children were excluded if they were outside the specified age range, had developmental dental anomalies, or were physically and/or mentally disabled. Additionally, children were excluded if their parents or guardians declined to participate in the survey.

Ethical considerations

Prior to participation, informed consent was obtained from the parents or guardians of all eligible children. The purpose and procedures of the study were clearly explained, and confidentiality was assured. Ethical approval was obtained from the Institutional Ethics Committee of Rajasthan University of Health Sciences College of Dental Sciences and Hospital (approval no. EC/PG-07/2021).

The distribution of study subjects is shown in Table 1.

**Table 1:** Age-wise distribution of children aged 3-6 years included in the study and the prevalence of Early Childhood Caries (ECC).

Study Subjects	N	Percentage
Age in years		
3 years	64	24.6
4 years	59	22.7
5 years	72	27.7
6 years	65	25.0
Caries Status		
ECC Absent	170	65.3
ECC Present	90	34.6

Questionnaire

The questionnaire, available in both Hindi and English based on the respondents’ language preference, was administered to parents accompanying their children to the hospital OPD and completed on the same day in the waiting area (see Appendices). It was self-designed by the primary investigator following an extensive literature review on early childhood caries and its associated risk factors, and its content validity was reviewed by three senior faculty members in pediatric dentistry to ensure clarity, relevance, and appropriateness. Developed in English and Hindi using a forward-backward translation method with expert review to ensure semantic equivalence, the tool was pilot-tested on 26 participants (10% of the sample) and demonstrated good internal consistency with a Cronbach’s alpha of 0.84. The questionnaire gathered comprehensive information on sociodemographic characteristics (child’s age, sex, ethnicity, place of residence, gestational age, family size, parental education and income, and caregiver dental health status), dietary practices (feeding methods, frequency of sweet consumption, and eating before bedtime), and oral hygiene behaviors (age at initiation of tooth brushing, brushing frequency, parental supervision during brushing, and history of dental visits or parental oral health guidance) [3].

Clinical examination

The clinical examination was conducted by the first author (Surbhi Sharma), a postgraduate trainee in Pedodontics, under the supervision of a senior faculty member. All assessments were performed by a single calibrated examiner to ensure consistency, with calibration established through pre-study inter- and intra-examiner reliability testing using Cohen’s Kappa, which yielded a value of 0.87, indicating excellent agreement. Dental examinations were carried out with the child seated upright on a standard dental chair, using a plain mouth mirror and a Community Periodontal Index (CPI) probe, with all instruments sterilized in an autoclave prior to use. Dental caries were diagnosed based on the decayed, missing, and filled teeth indices (DMFT/deft), following the World Health Organization (WHO, 1997) guidelines [15].

Socioeconomic status (SES)

The Modified Kuppuswamy Scale assessed the SES. The modified Kuppuswamy scale is a widely used tool for evaluating socioeconomic status (SES) in both urban and rural settings. Introduced by Kuppuswamy in 1976, the scale calculates a composite score ranging from 3 to 29, based on three key factors: the educational level and occupation of the head of the family, as well as the family's monthly income. This scale classifies the study populations into five SES, as shown in Table 2.

**Table 2:** Modified Kuppuswamy scale (update for February 2019) The table scores the head of the household's education (1–7), occupation (1–10), and monthly income (1–12, adjusted to Feb 2019 CPI). The total score (range: 3–29) determines the socioeconomic class, from Class I (Upper) to Class V (Lower). Higher scores indicate better socioeconomic standing. SES: socioeconomic status Source: Wani (2019) [10].

Component	Criteria	Score
Education	Professional degree	7
	Graduate or postgraduate	6
	Intermediate or post high school diploma	5
	High school certificate	4
	Middle school certificate	3
	Primary school certificate	2
	Illiterate	1
Occupation	Professional (white collar)	10
	Semi-professional	6
	Clerical, shop-owner/farmer	5
	Skilled worker	4
	Semi-skilled worker	3
	Unskilled worker	2
	Unemployed	1
Monthly Income (Feb 2019 CPI)	≥ ₹52,734	12
	₹26,355–₹52,733	10
	₹19,759–₹26,354	6
	₹13,161–₹19,758	4
	₹7,887–₹13,160	3
	₹2,641–₹7,886	2
	≤ ₹2,640	1
SES Class	26–29 – Upper (Class I)	
	16–25 – Upper Middle (Class II)	
	11–15 – Lower Middle (Class III)	
	5–10 – Upper Lower (Class IV)	
	01–04 – Lower (Class V)	

 Statistical analysis

All collected data were entered into Microsoft Excel 2007 (Microsoft Corporation, Redmond, USA) and analyzed using SPSS Statistics version 23.0 (IBM Corp., Armonk, USA). The statistical significance threshold was set at p < 0.05.

Nature of Data

All primary study variables, such as socioeconomic status, dietary habits, oral hygiene practices, and caries presence, were categorical. Therefore, parametric tests requiring assumptions of normality were not applicable. No continuous variables were included in the core analysis, and hence, normality testing (e.g., Shapiro-Wilk test) was not conducted.

Descriptive Statistics

Descriptive statistics were used to summarize the data. Frequencies and percentages were calculated for all categorical variables (e.g., age groups, brushing frequency, feeding type, socioeconomic class, etc.). Cross-tabulations were used to explore relationships between caries status and independent variables.

Test of Association

To determine the statistical association between Early Childhood Caries (ECC) and various independent variables, the Pearson’s Chi-square test of independence was employed. This test was applied to 2x2 and RxC contingency tables where all expected cell counts were ≥5. For variables where any expected cell count was <5, Fisher’s Exact Test was considered, although not required in this dataset.

The Chi-square test was used to evaluate associations with: a. Socioeconomic status; b. Feeding practices; c. Brushing behavior; d. Demographic variables (e.g., gender, number of siblings); and e. Parental oral health status.

Software

All analyses were performed using SPSS v23.0. No imputation was done for missing data, and only complete responses were included in the final analysis.

## Results

Non-significant results were obtained for ECC in its association with the age and gender of the children (Table 3). 

**Table 3:** Early childhood caries (ECC) distribution by age and gender The Chi-square test at a p-value < 0.05 is considered significant.

Description	Category	Total (%)	Children with ECC (%)	Chi-square Value	p-value	Significance
Age	3 years	24.6	32.8	2.614	0.454	Non-significant
	4 years	22.7	42.4			
	5 years	27.7	29.2			
	6 years	25.0	33.8			
Gender	Male	58.5	34.9	0.065	0.874	Non-significant
	Female	41.5	33.3			

When the prevalence of ECC was analyzed by socioeconomic status, children with a lower socioeconomic status (66.7%) were more affected, followed by middle socioeconomic status (60.1%), and the least affected were children of upper socioeconomic status (6.8%) (Table 4).

**Table 4:** Prevalence of ECC in children according to the socioeconomic status of the family The Chi-square test at a p-value < 0.05 is considered significant. ECC: early childhood caries

SES Category	Total (%)	ECC (%)	Chi-square Value	p-value	Significance
Upper	22.7	6.8	51.835	0.001	Significant
Upper-middle	29.6	32.5			
Lower-middle	22.3	27.6			
Upper-lower	25.4	66.7			
Lower	0.0	0.0			

The occurrence of ECC was notably lower among children who began tooth brushing at or before the age of three. Conversely, a delayed start in tooth brushing was associated with a higher prevalence of ECC. Additionally, a greater frequency of tooth brushing was significantly linked to an increased prevalence of ECC. However, no significant association was observed between ECC prevalence and parental supervision of tooth brushing (Table 5).

**Table 5:** Association of ECC with oral hygiene behavior The Chi-square test at a p-value < 0.05 is considered significant. ECC: early childhood caries

Variable	Category	Total (%)	ECC (%)	Chi-square Value	p-value	Significance
Start Tooth Brushing	≤3 years	52.3	11.6	21.513	0.001	Significant
	4 years	23.8	20.6			
	5 years	9.2	31.7			
	Never	14.6	43.7			
Daily Brushing Frequency	Never/Infrequent	26.5	46.4	30.021	0.001	Significant
	Once daily	44.2	33.5			
	>Once daily	29.2	29.2			
Parental Supervision of Brushing	Yes	62.7	33.7	0.045	0.465	Non-significant
	No	37.3	35.1			

The study found no significant link between ECC prevalence and birth weight or delivery type. There was a direct association between the prevalence of ECC and the number of siblings. That implies the more the number of siblings in the family, the more the prevalence of ECC. A significant result was obtained between the prevalence of ECC and parental caries (Table 6).

**Table 6:** Association of ECC with socio-demographic variables The Chi-square test at a p-value < 0.05 is considered significant. ECC: early childhood caries

Variable	Category	Total (%)	ECC (%)	Chi-square Value	p-value	Significance
Birth Weight	Low	18.0	32.7	0.087	0.868	Non-significant
	Normal	82.0	34.8			
Type of Delivery	Normal	63.5	34.5	0.020	0.967	Non-significant
	Cesarean	36.5	33.7			
Siblings	One child	15.8	26.6	3.456	0.045	Significant
	>One child	84.2	35.6			
Parental Caries	Yes	48.1	40.0	3.540	0.040	Significant
	No	51.9	28.9			

A higher prevalence of ECC was observed in children who practiced mainly bottle feeding at night, often practiced eating before sleep, and had a sweet intake once or more daily (Table 7).

**Table 7:** Association of ECC with feeding practice The Chi-square test at a p-value < 0.05 is considered significant. ECC: early childhood caries

Variable	Category	Total (%)	ECC (%)	Chi-square Value	p-value	Significance
Manner of Feeding	Only breastfed	33.5	17.2	23.508	0.001	Significant
	Both breast & bottle	44.6	36.2			
	Only bottle-fed	21.9	56.2			
Eating Before Sleep	Often	34.2	43.9	15.340	0.001	Significant
	Sometimes	25.4	43.9			
	No	40.5	20.0			
Sweet Intake Frequency	Several times/month	33.5	23.1	13.113	0.001	Significant
	Several times/week	26.5	27.6			
	Once or more daily	40.0	47.2			

## Discussion

The present study assessed the prevalence and determinants of Early Childhood Caries (ECC) among children aged 3-6 years in Jaipur, Rajasthan, identifying a prevalence of 34.6%. This rate is lower than findings from prior Indian studies that have reported an ECC prevalence ranging from 40.4% to 81% in various regions. Sonika et al. observed higher rates in North India, suggesting possible regional disparities related to dietary, cultural, and socioeconomic variations [6].

A significant association was observed between socioeconomic status (SES) and ECC. Children from lower SES groups exhibited a higher prevalence (66.7%) of ECC, corroborating findings from international contexts such as Lithuanian cities and Ludhiana city, where children from disadvantaged backgrounds had significantly more dental decay [16,17]. These results are in line with those reported by Lagreca and Boustedt et al., who noted increased ECC incidence in lower-income populations due to poor access to dental care and oral health education [18,19].

Feeding practices emerged as significant predictors of ECC. Children who were exclusively bottle-fed or combined breast and bottle feeding demonstrated higher caries rates, consistent with prior studies that identified prolonged bottle feeding as a risk factor for ECC. Anil and Anand and Valaitis et al. similarly noted the cariogenic potential of extended bottle-feeding and nighttime feeding behaviors [5, 20]. Furthermore, frequent sweet consumption and eating before sleep significantly increased ECC risk, affirming the role of cariogenic diets as established in multiple studies, including that of Elamin et al. [3].

Oral hygiene behaviors also showed significant associations. Early initiation of brushing (before 3 years of age) and brushing frequency were protective against ECC, consistent with the findings of Cascaes et al. and Boustedt et al. [19,12]. However, unlike some literature that supports supervised brushing as a preventive measure (e.g., Simratvir et al., 2009 [17]), this study did not find a significant association between parental supervision and ECC prevalence.

Among socio-demographic variables, the number of siblings was positively correlated with ECC prevalence. This finding supports earlier work by Knoblauch et al., suggesting that larger family size may dilute parental attention to individual children’s oral health [8]. Similarly, parental caries status was significantly associated with ECC in their children, reinforcing the intergenerational transmission of oral health behaviors and bacterial flora, as emphasized by Isong et al. [13].

Conversely, no significant relationship was found between ECC and birth weight, type of delivery, or gender - findings that align with previous research by Costa et al. and Singh et al., which showed inconsistent or minimal associations between these variables and ECC [14,21].

Collectively, this study reinforces ECC as a multifactorial condition shaped by socioeconomic, behavioral, and familial factors. The alignment of findings with prior literature underscores the global consistency of ECC determinants while highlighting the urgent need for targeted public health strategies in vulnerable populations.

Limitations

While this study sheds light on the relationship between socioeconomic factors and early childhood caries (ECC), a few limitations must be acknowledged. Being cross-sectional in design, it captures associations at a single point in time, making it difficult to determine cause and effect. The use of convenience sampling from a single center may limit how well the results apply to the general population. Some information, such as feeding habits and brushing routines, was based on parents’ recall, which may not always be accurate. Also, factors like access to dental care, parental oral health knowledge, and fluoride exposure were not considered and could have influenced the outcomes. Future research with broader, more diverse samples and longitudinal follow-up would help validate and expand on these findings.

Clinical implications

This study underscores the strong association between socioeconomic status and early childhood caries (ECC), highlighting the need for targeted preventive strategies in lower-income groups. Public health programs should prioritize oral health education, early intervention, and increased access to pediatric dental care for underserved populations.

Findings related to feeding practices, sweet intake, and delayed tooth brushing reinforce the importance of educating caregivers on proper dietary and oral hygiene habits from infancy. The link between parental and child caries also supports a family-centered approach to oral health promotion.

Overall, integrating ECC prevention into maternal and child health services, with a focus on behavioral and socioeconomic factors, is essential for reducing the burden of ECC.

Future recommendations

This study emphasizes the influence of socioeconomic and behavioral factors on early childhood caries (ECC). Future research should focus on longitudinal studies to establish causal relationships and evaluate the long-term impact of interventions. Community-based oral health education programs targeting high-risk, low socioeconomic groups are recommended, along with the integration of oral health into maternal and child health services.

Further studies with larger, more diverse populations across different regions would enhance generalizability. Additionally, school-based preventive programs and randomized trials should be explored to identify effective, scalable strategies for ECC prevention. A multidisciplinary, policy-driven approach is essential to reduce disparities and improve pediatric oral health outcomes.

## Conclusions

The present study underscores the multifactorial etiology of early childhood caries (ECC), with a particular emphasis on the influence of socio-demographic and behavioral determinants. Children from lower socioeconomic backgrounds were found to be more susceptible to ECC, with factors such as parental education, occupation, and family income serving as significant indicators. A positive association was observed between the number of siblings and caries incidence, suggesting that familial dynamics may impact oral health outcomes. Furthermore, early initiation and increased frequency of tooth brushing were associated with a reduced risk of ECC, reinforcing the importance of oral hygiene practices from an early age. Dietary behaviors also played a critical role; children who were exclusively bottle-fed, consumed sweets frequently between meals, and had a habit of eating before sleep exhibited higher caries prevalence. Additionally, a positive correlation was found between caregivers with existing dental caries and the caries status of their children, highlighting the potential influence of caregiver oral health on pediatric dental outcomes. Notably, no significant associations were observed between ECC occurrence and parental supervision, type of delivery, or birth weight. These findings highlight the need for comprehensive preventive strategies that address socioeconomic disparities, caregiver education, dietary habits, and oral hygiene practices to effectively manage and reduce the burden of ECC.

## Appendices

Questionnaire of the study  

**Table 8:** Patient Details

Question	Response Options
Patient Name	
OPD No.	
Age of Patient	3 / 4 / 5 / 6 years
Gender of Patient	Male / Female

**Table 9:** Socioeconomic Status

Question	Response Options
Education of Head of Family	Professional / Graduate / Intermediate / High school / Middle school / Primary / Illiterate
Occupation of Head of Family	Professional / Semi-professional / Clerical / Skilled / Semi-skilled / Unskilled / Unemployed
Monthly Income (Feb 2019 CPI)	≥52,734 / 26,355–52,733 / 19,759–26,354 / 13,161–19,758 / 7,887–13,160 / 2,641–7,886 / ≤2,640

**Table 10:** Medical History

Question	Response Options
History of Chronic Illness in Parents	Yes / No
If Yes, Specify	
Any Systemic Disease to Child	Yes / No
If Yes, Specify	

**Table 11:** Demographic Information

Question	Response Options
Residency	Urban / Rural
School Type	Public / Private
Type of Delivery	Normal / Forcep / Cesarean
Birth Weight	Low / Normal / Overweight
Gestational Age of Child	Full term / Premature / Post term
Family Size	One child / More than one child
Caretaker has Caries or Gum Disease	Yes / No

**Table 12:** Dietary Behaviour

Question	Response Options
Feeding History	Breast only / Both / Bottle only
Age of Weaning	≤6 months / 6–12 months / >12 months
Eating Before Sleep	Often / Sometimes / No
Sweet Intake Frequency	Several times/month / week / daily

**Table 13:** Oral Hygiene Behaviour

Question	Response Options
Start Tooth Brushing	≤3 years / 4 years / 5 years / Never
Daily Brushing Frequency	Never / Once / More than once
Parental Supervision of Brushing	Yes / No
Parents’ Perception of Child’s Dental Status	Very good / Satisfactory / Unsatisfactory / Very unsatisfactory

**Table 14:** Use of Dental Services

Question	Response Options
Dental Visit in the Past	Yes / No
Parents Received Oral Health Instructions	Yes / No
History of Dental Treatment of Parents	Yes / No

अध्ययन की प्रश्नावली    

**Table 15:** मरीज़ का विवरण

प्रश्न	उत्तर विकल्प
मरीज़ का नाम	
ओपीडी संख्या	
मरीज़ की आयु	3 / 4 / 5 / 6 वर्ष
लिंग	पुरुष / महिला

**Table 16:** सामाजिक-आर्थिक स्थिति

प्रश्न	उत्तर विकल्प
परिवार के मुखिया की शिक्षा	प्रोफेशनल / स्नातक / इंटरमीडिएट / हाई स्कूल / मिडिल स्कूल / प्राइमरी / निरक्षर
परिवार के मुखिया का व्यवसाय	प्रोफेशनल / अर्ध-प्रोफेशनल / क्लर्क / कुशल / अर्ध-कुशल / अकुशल / बेरोज़गार
मासिक आय (फरवरी 2019 CPI)	≥52,734 / 26,355–52,733 / 19,759–26,354 / 13,161–19,758 / 7,887–13,160 / 2,641–7,886 / ≤2,640

**Table 17:** चिकित्सा इतिहास

प्रश्न	उत्तर विकल्प
माता-पिता में पुरानी बीमारी का इतिहास	हाँ / नहीं
यदि हाँ, तो कृपया बताएं	
क्या बच्चे को कोई प्रणालीगत बीमारी है	हाँ / नहीं
यदि हाँ, तो कृपया बताएं	

**Table 18:** जनसांख्यिकीय जानकारी

प्रश्न	उत्तर विकल्प
निवास स्थान	शहरी / ग्रामीण
विद्यालय का प्रकार	सरकारी / निजी
प्रसव का प्रकार	सामान्य / फोर्सेप / सिज़ेरियन
जन्म का वजन	कम / सामान्य / अधिक
गर्भकाल की अवधि	पूर्ण अवधि / समयपूर्व / बाद अवधि
परिवार का आकार	एक बच्चा / एक से अधिक बच्चे
क्या देखभालकर्ता को दांतों या मसूड़ों की बीमारी है	हाँ / नहीं

**Table 19:** आहार व्यवहार

प्रश्न	उत्तर विकल्प
खिलाने का इतिहास	केवल स्तनपान / दोनों / केवल बोतल
स्तनपान छुड़ाने की आयु	≤6 महीने / 6–12 महीने / >12 महीने
सोने से पहले भोजन	अक्सर / कभी-कभी / नहीं
मिठाई सेवन की आवृत्ति	प्रति माह कई बार / प्रति सप्ताह / प्रतिदिन

**Table 20:** मौखिक स्वच्छता व्यवहार

प्रश्न	उत्तर विकल्प
ब्रश करना कब शुरू किया	≤3 वर्ष / 4 वर्ष / 5 वर्ष / कभी नहीं
दैनिक ब्रशिंग की आवृत्ति	कभी नहीं / एक बार / एक से अधिक बार
ब्रशिंग पर माता-पिता की निगरानी	हाँ / नहीं
बच्चे के दंत स्वास्थ्य पर माता-पिता की धारणा	बहुत अच्छा / संतोषजनक / असंतोषजनक / बहुत असंतोषजनक

**Table 21:** दंत सेवाओं का उपयोग

प्रश्न	उत्तर विकल्प
क्या पहले कभी दंत चिकित्सक के पास गए	हाँ / नहीं
क्या माता-पिता को मौखिक स्वास्थ्य निर्देश प्राप्त हुए	हाँ / नहीं
माता-पिता के दंत उपचार का इतिहास	हाँ / नहीं
